# Supplementary material for: The Streptococcus agalactiae R3 surface protein is encoded by sar5
Source: PLoS One. 2022 Jul 29;17(7):e0263199. doi: 10.1371/journal.pone.0263199 (PMC9337641; doi:10.1371/journal.pone.0263199)

S1 Supporting Materials and Methods.

To create pKT1-sar5-F and pKT1-sar5D-F, the backbone of pKT1 was amplified using primers:

pKT1_vector_F (Vector.FOR):
AATGTAACTGTATTCAGCGATGACGAAATTCT
pKT1_vector_R (Vector.REV):
ATGTTCATGACTCCATTATTATTGTACATGTTGCA

To create pKT1-sar5-F, the FLAG-tagged sar5 was amplified from GBS strain using primers:

pKT1_sar5F_F:
CAATAATAATGGAGTCATGAACATATGTTTAGTCAATATAATTTTGAAAAAGGTTTAAATTTTTCCAT
pKT1_sar5F_R - (FLAG-tag in red):
ATCGCTGAATACAGTTACATTTTACTTGTCGTCATCGTCTTTGTAGTCATTTTTACGTCGTTTTAAACTGCTAGCTGC

To create pKT1-sar5D-F, the FLAG-tagged sar5D was amplified using primers:

pKT1_sar5D-F_F:
CAATAATAATGGAGTCATGAACATATGTTTCGTAAATATAATTTTGAAAAAGGTTTAAAGTTTTCCAT
pKT1_sar5F_R - (FLAG-tag in red):
ATCGCTGAATACAGTTACATTTTACTTGTCGTCATCGTCTTTGTAGTCATTTTTACGTCGTTTTAAACTGCTAGCTGC

The PCR amplified products were adjoined by Gibson assembly.

Cloning strategy of pKT1-sar5-F:

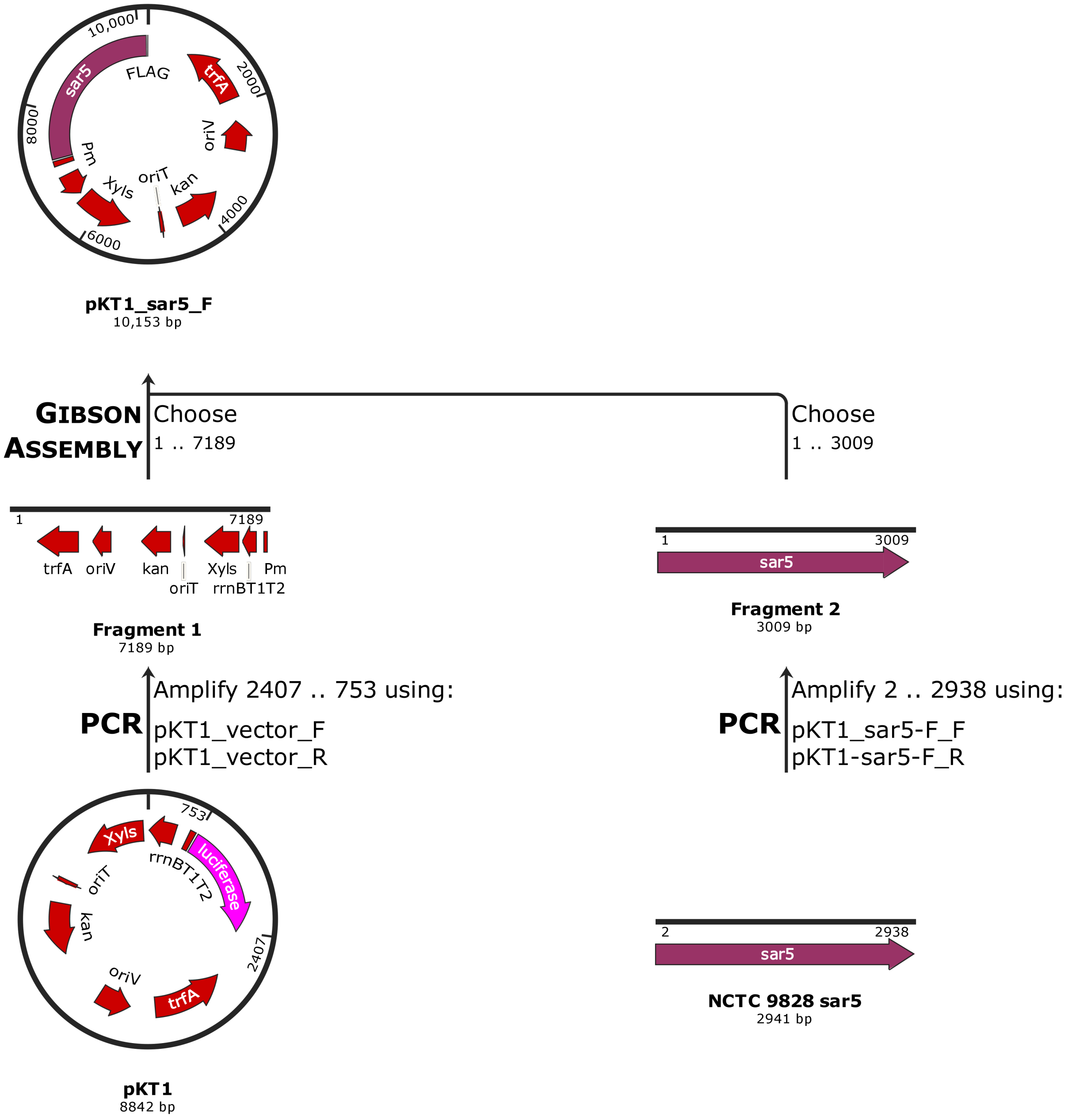

Cloning strategy of pKT1-sar5-F:

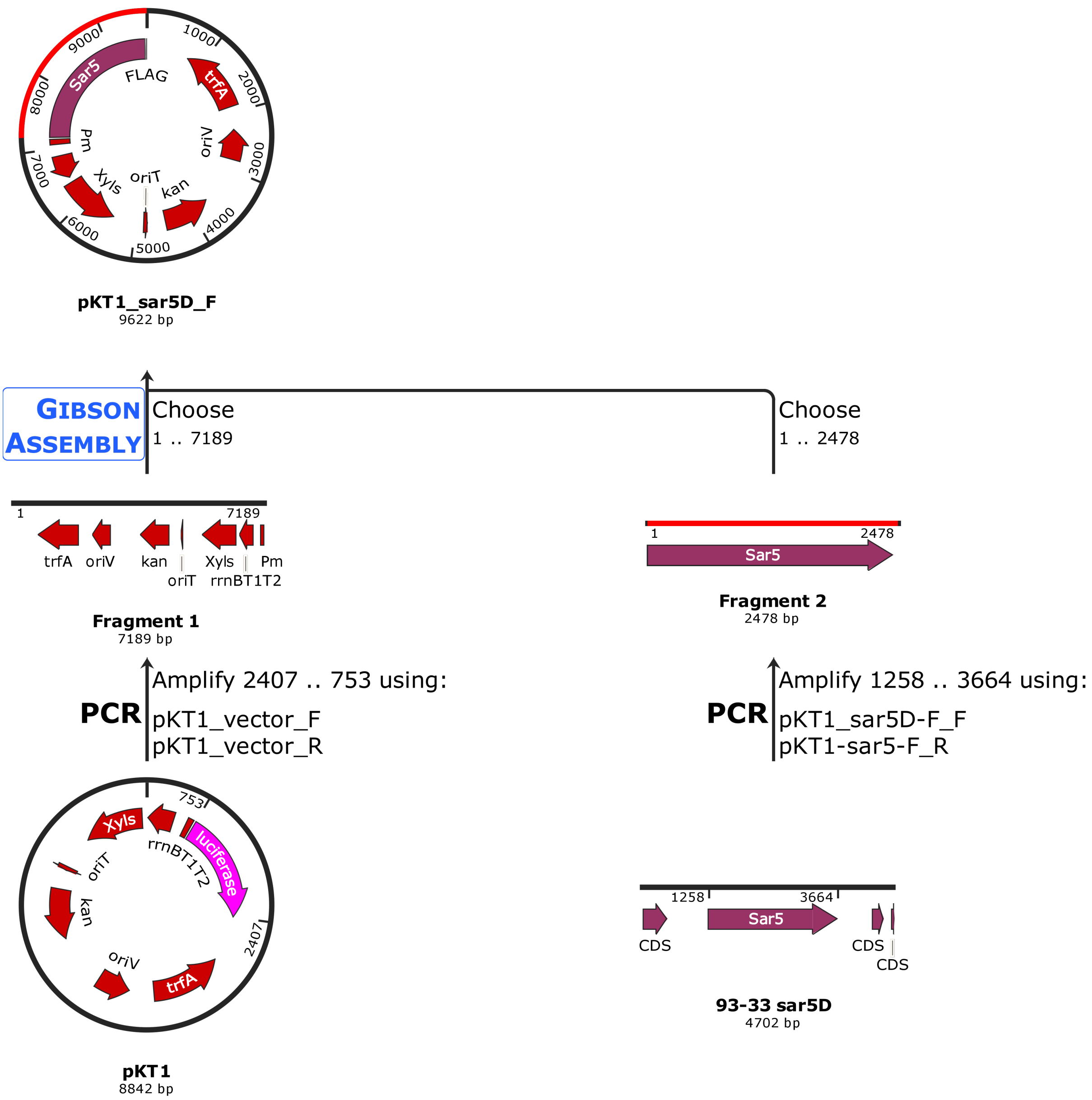

Supplement: S1 File — (PDF) [file pone.0263199.s002.pdf]
