# Supplementary material for: The Streptococcus agalactiae R3 surface protein is encoded by sar5
Source: PLoS One. 2022 Jul 29;17(7):e0263199. doi: 10.1371/journal.pone.0263199 (PMC9337641; doi:10.1371/journal.pone.0263199)

A

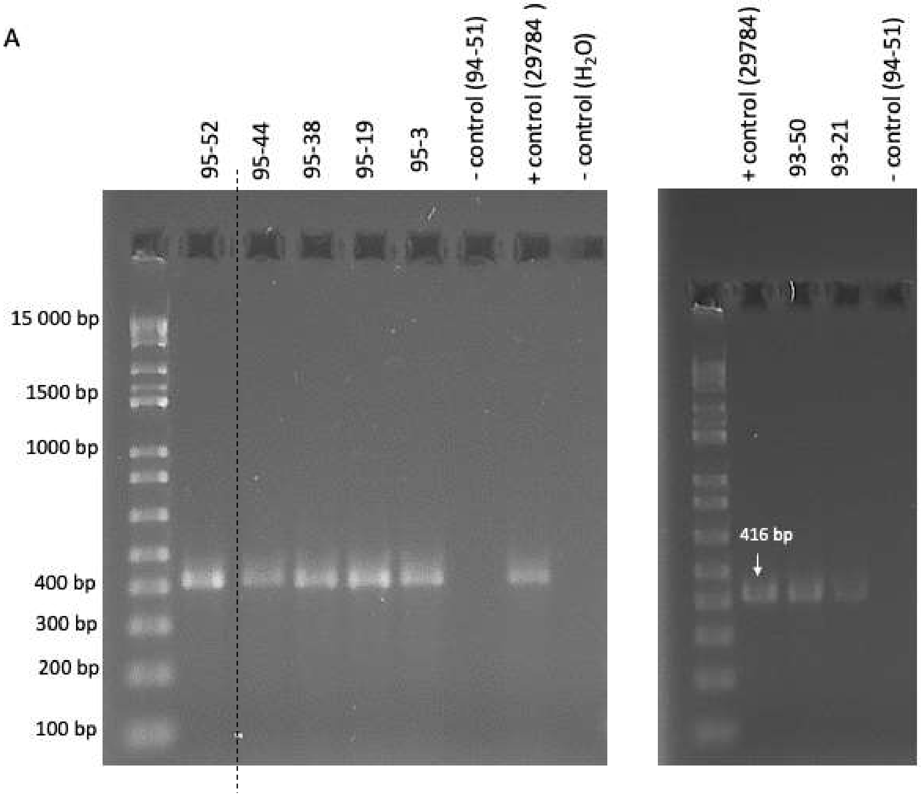

B

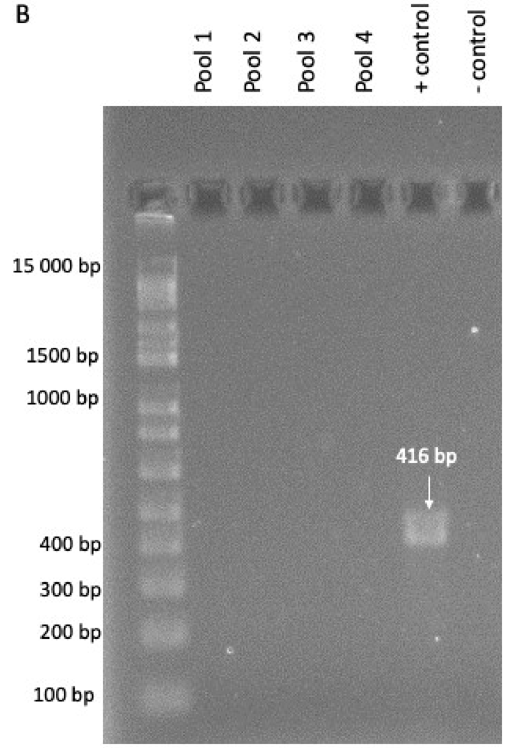

Pool 1= 94-30, 94-38, 94-13

Pool 2= 94-19, 94-24, 94-26

Pool 3= 94-55, 94-1, 94-14

Pool 4= 95-9, 95-39, 95-64

C

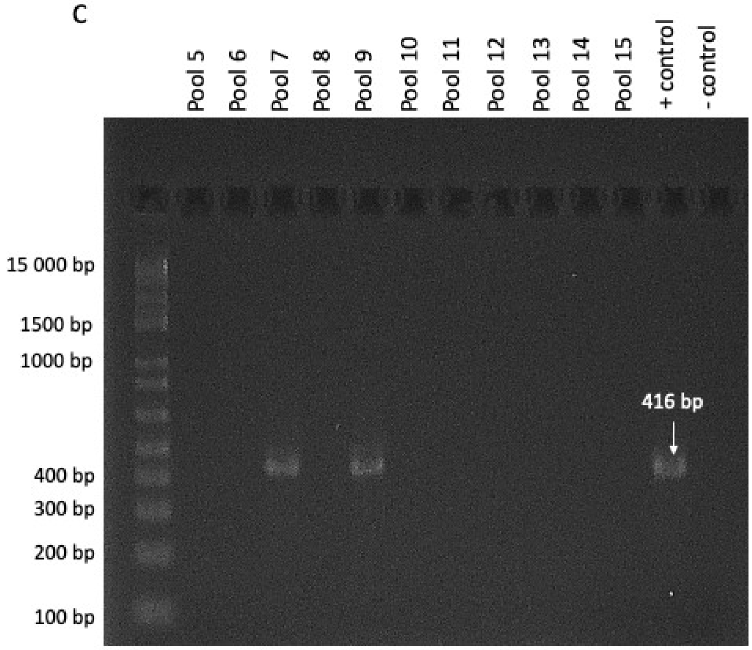

Pool 5= 92-3, 92-4, 93-7, 93-8, 93-48

Pool 6= 93-14, 93-16, 93-17, 93-18, 92-2

Pool 7= 93-10, 93-11, 92-1, 93-33, 93-29

Pool 8= 93-13, 92-5, 92-6, 93-28, 93-30

Pool 9= 93-32, 94-3, 94-4, 94-7, 94-10

Pool 10= 94-11, 94-18, 94-23, 94-28, 94-31

Pool 11= 94-39, 94-40, 94-42, 94-46, 94-48

Pool 12= 94-50, 94-56, 95-6, 95-7, 95-8

Pool 13= 95-12, 95-14, 95-16, 95-23, 95-26

Pool 14= 95-30, 95-33, 95-34, 95-36, 95-37

Pool 15= 95-40, 95-41, 95-42, 95-43, 95-45

D

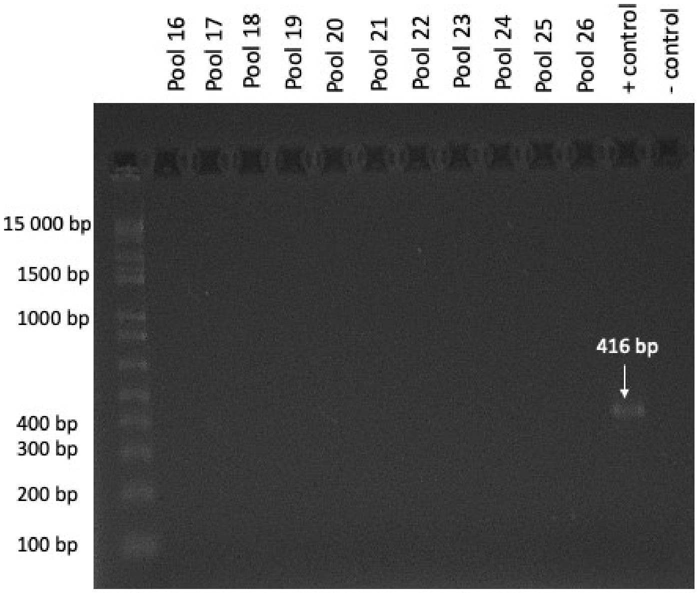

Pool 16= 95-48, 95-49, 95-53, 95-54, 95-59

Pool 17= 95-60, 95-61, 95-62, 95-65, 95-66

Pool 18= 95-63, 94-2, 94-12, 94-17, 94-20

Pool 19= 94-21, 94-22, 94-25, 94-27, 94-29

Pool 20= 94-32, 94-33, 94-34, 94-35, 94-41

Pool 21= 94-44, 94-52, 94-53, 94-54, 95-2

Pool 22= 95-5, 95-10, 95-11, 95-13, 95-21

Pool 23= 95-22, 95-27, 95-28, 95-29, 95-31

Pool 24= 95-47, 94-21484, 94-6, 94-8, 94-9

Pool 25= 94-36, 94-47, 94-49, 94-57, 95-1

Pool 26= 95-4, 95-15, 95-25, 95-35, 95-58

E

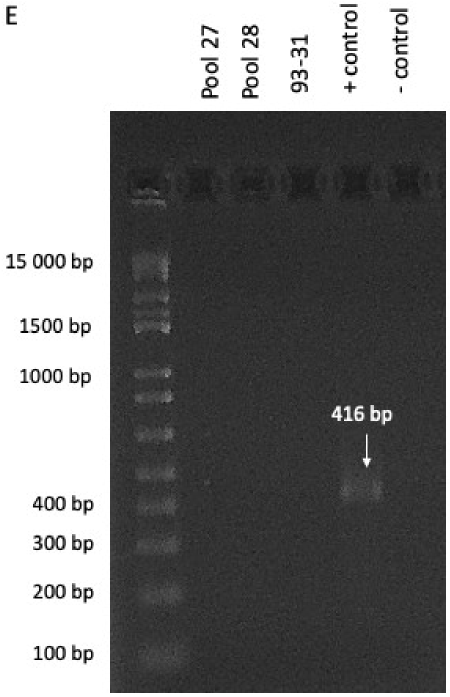

Pool 27= 94-15, 94-37, 94-45, 95-24, 94-43

Pool 28= 93-22, 93-23, 93-24, 93-25, 93-27

F

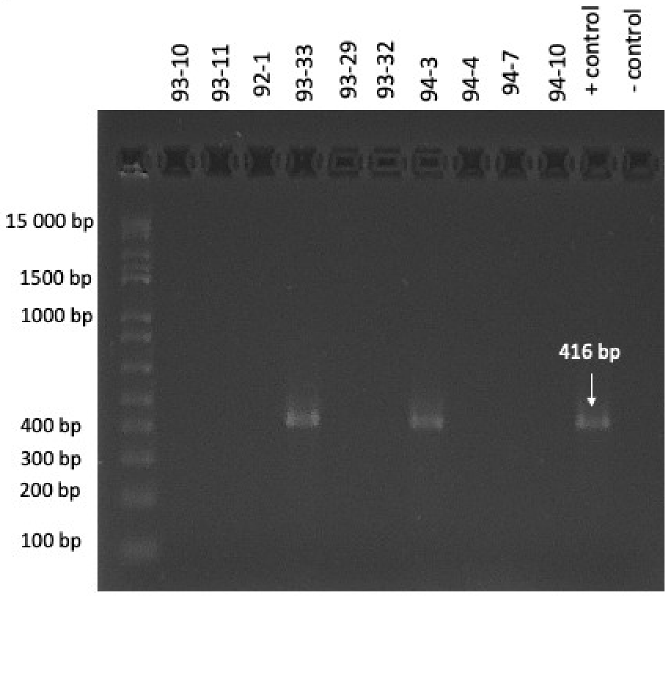

Supplement: S1 Fig — The black dashed line indicates that the image was edited at this location, removing one lane containing a sample that was excluded from the collection due to uncertainties around its identity. B-E) amplification of sar5 from the 133 R3 negative strains, in pools of 3–5 strains. F) amplification of sar5 from the strains within pool 7 and pool 9. Raw data images of electrophoresis gels are found in S1 Raw images. (PDF) [file pone.0263199.s003.pdf]
