# Supplementary material for: The Streptococcus agalactiae R3 surface protein is encoded by sar5
Source: PLoS One. 2022 Jul 29;17(7):e0263199. doi: 10.1371/journal.pone.0263199 (PMC9337641; doi:10.1371/journal.pone.0263199)
Supplement: S2 Fig — Alignments were made using SnapGene software (from Insightful Science). (PDF) [file pone.0263199.s004.pdf]

S2 Supplementary

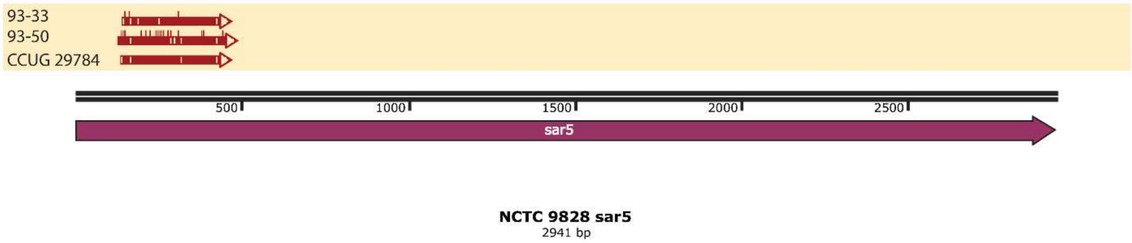

S2 Figure: Alignment of sequenced *sar5* PCR products of CCUG 29784, 93-33, and 94-3 to the *sar5* gene of the whole genome sequenced strain NCTC 9828. Alignments were made using SnapGene software (from Insightful Science).

Below are the sequences:

93-33:
CTAGTGTTACATTCTGGCAACACACTCCAACAGGTGTAACGGCAACAGATGCAAACTTG
GTCAATCCTAACAATTACAACTCCTACTTACCACTAATAGGAGTCGCAACTAGCACTCAA
GGTAAGTCAATTTCGAGTCAATCACTTACAGAAATCATTAAGCCTCGCAACTCCCAGCAG
CAACAACACCAACAACTCGATAATGCAGCCCCATCAGTAGACAAGAGGACGTATGCTAC
TAGTGGCGATTGGACGTTACAAAATCCATATCGCTCGACAGTGTTCGAAATAAAAATATT
TCTCCAAGTGTTCGTGATGAATCATTTAAAAGTCGCTCGAAACAACGTGGGTGTTTCTCA
AAAA

93-50:
ACTAGCAACAACTCCAACAGGTGTAACGGCAACAGATGCAAACTTGGTCAATCCTAACA
ATTCAACTCCTACTTCCACTAATAGGAGTGCAACTAGCACTCAAGGAAGTAATTTGAGTA
ATACTTCAGAAATCATTAAGCCTGCAACTTTAGCAGCAACATCACCAACAACTGATAATG
CAGCCCCATCAGTAGACAAGAGGACGTATGCTACTAGTGGCGATTGGACGTTACAAAAT
CCATATGCTGACAGTGTTCGAAATAAAAATATTTCTCCAAGTGTTCGTGATGAATCATTT
AAAAGTGCTGAAACACGAGGGGTTTTCGCAAAA

CCUG 29784:
TGGCAACAACTTCCAACAGGTGTAAACGGCCACAGATGCAAACTTGGTCACTTCTAACA
ATTCAACTCCTACTTCCACTAATAGGAGTGCAACTAGCACTCAAGGAAGTAATTCGAGTA
ATACTTCAGAAATCATTAAGCCTGCAACTTTAGCAGCAACATCACCAACAACTCGATAAT
GTAGCCCCATCAGTAGACAAGAGGACGTATGCTACTAGTGGCGATTGGACGTTACAAAA
TCCATATGCTGACAGTGTTCGAAATAAAAATATTTCTCCAAGTGTTCGTGATGAATCATT
TAAAAGTGCTGAACACGTAGGTTTTTCTCAAAAC
