# Supplementary material for: The Streptococcus agalactiae R3 surface protein is encoded by sar5
Source: PLoS One. 2022 Jul 29;17(7):e0263199. doi: 10.1371/journal.pone.0263199 (PMC9337641; doi:10.1371/journal.pone.0263199)

S1 Raw Images

Raw images for Figure 1C:

Order of loading:

1. Magic Marx XP Protein Standard (Invitrogen)
2. CCUG 29779
3. CCUG 29784
4. 93-33
5. 94-3

The images were captured using LI-COR Odyssey (LI-COR Biosciences)

GAPDH: 1 2 3 4 5

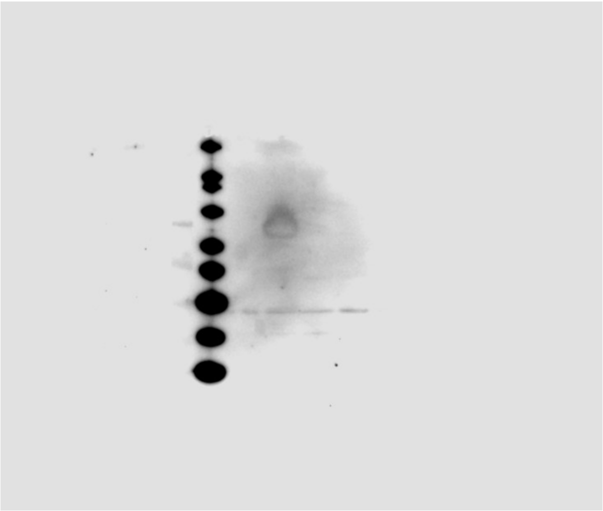

R3: 1 2 3 4 5 X

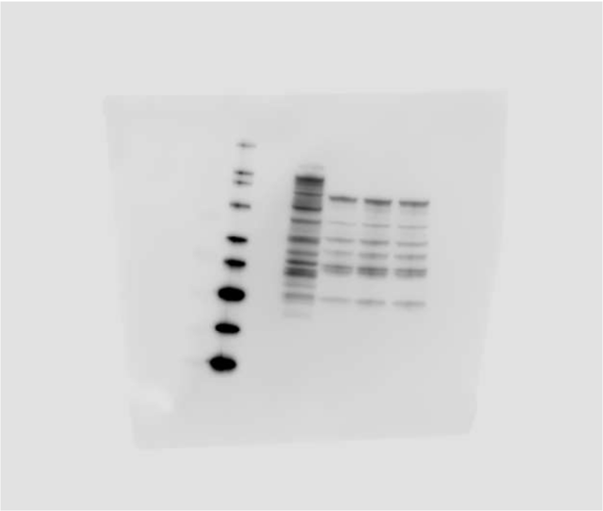

Raw images for Figure 4:

Order of loading:

1. pKT1-sar5D-F induced
2. pKT1-sar5D-F uninduced
3. pKT1-sar5-F induced
4. pKT1-sar5-F uninduced
5. pKT1 induced
6. pKT1 uninduced
7. Magic Marx XP Protein Standard (Invitrogen)

The images were captured using LI-COR Odyssey (LI-COR Biosciences)

R3: 1 2 3 4 5 6 7

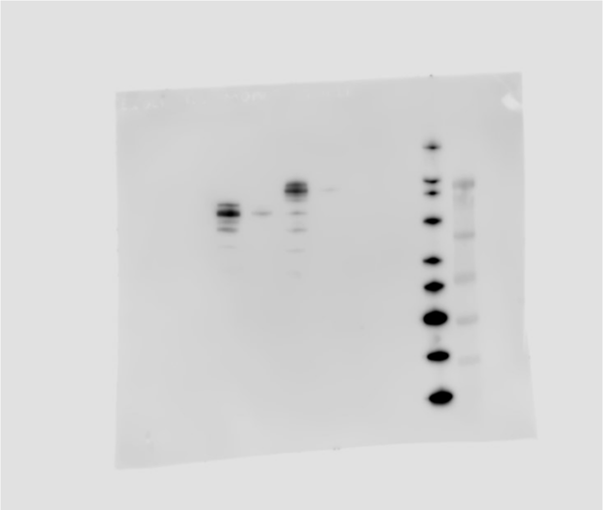

FLAG: 1 2 3 4 5 6 7

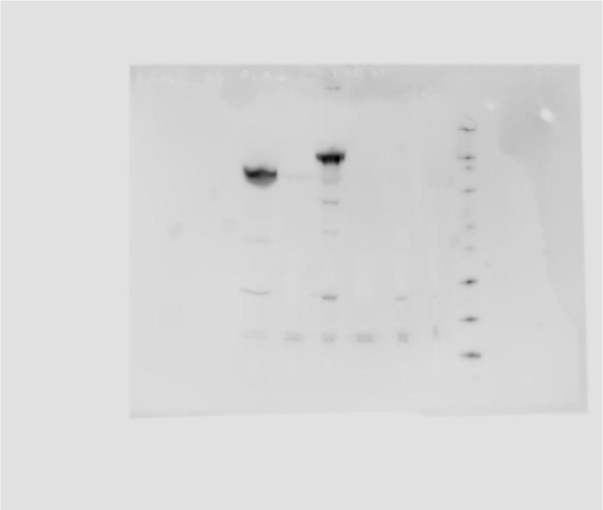

GAPDH: 7 6 5 4 3 2 1

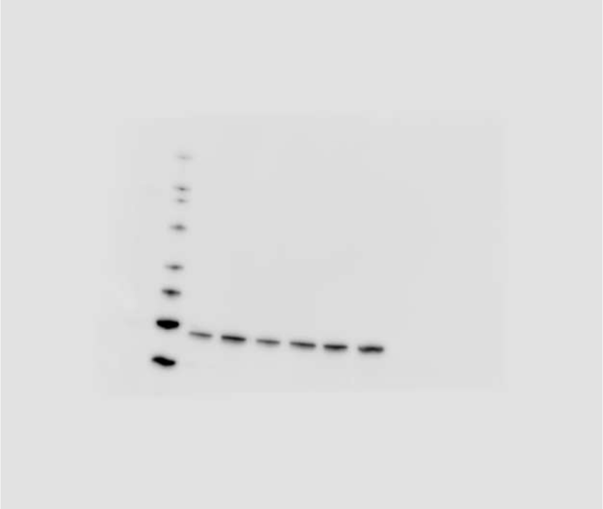

Raw images for S1

For Figure S1 A (right panel) and B-F the raw images are identical to those presented in the figure. For Figure S1 A (left panel), the raw image is found below.

Order of loading:

1. Generuler 1 Kb Plus DNA Ladder
2. 95-52
3. 95-51 (X)
4. 95-44
5. 95-38
6. 95-19
7. 95-3
8. 94-51 (negative control)
9. CCUG 29784 (positive control)
10. water (negative control)
11. Generuler 1 Kb Plus DNA Ladder

Sample number 3 (95-51) has been edited out in Figure S1. This sample was initially R3 positive, then later it tested R3 negative, and is sar5 negative. Because the initial R3 measurement was done in 1999 and there has likely been a mix up with this sample since then, we do not know which strain is now in the place of 95-51 and therefore found it best to omit this strain from our collection. The image editing is indicated in Figure S1A by a black dashed line and described in the corresponding figure legend.

1 2 3 4 5 6 7 8 9 10 11

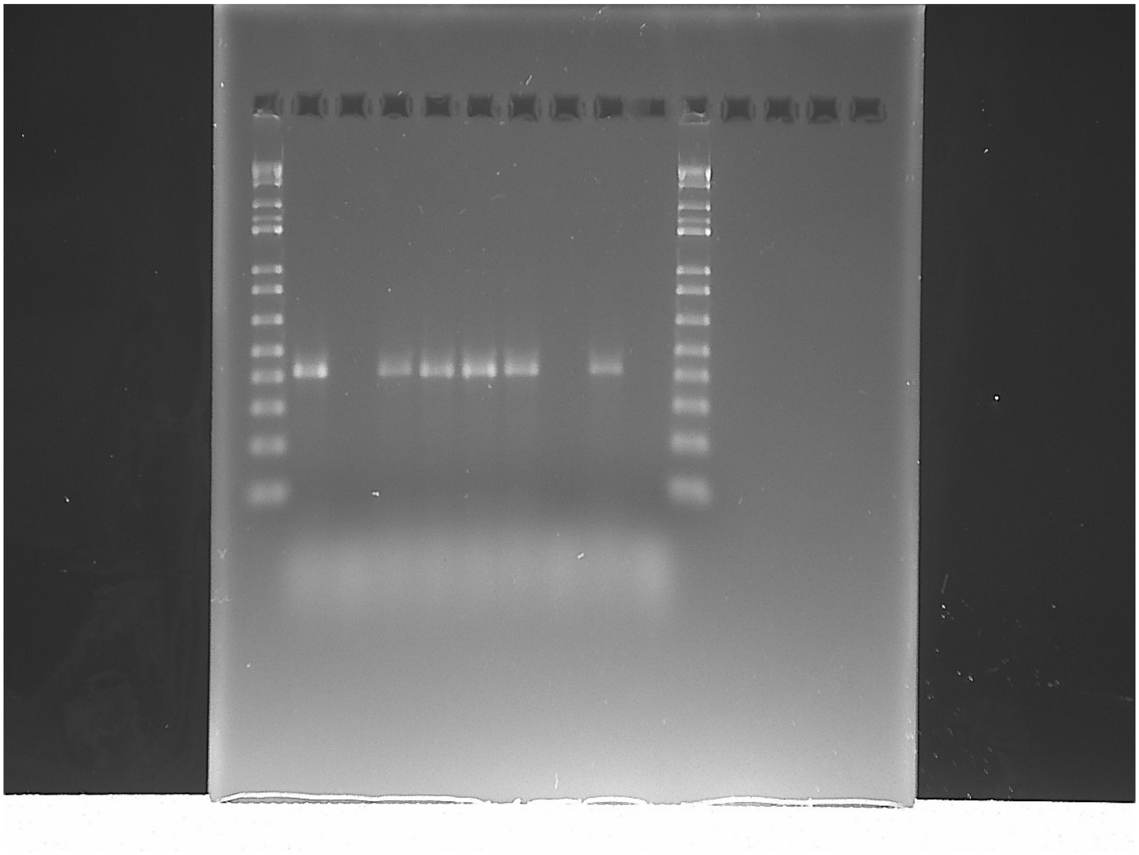

Supplement: S1 Raw images — (PDF) [file pone.0263199.s005.pdf]
